# The Effect of Mass Media Campaign on the Use of Insecticide-Treated Bed Nets among Pregnant Women in Nigeria

**DOI:** 10.1155/2014/694863

**Published:** 2014-03-20

**Authors:** A. Ankomah, S. B. Adebayo, E. D. Arogundade, J. Anyanti, E. Nwokolo, U. Inyang, Oladipupo B. Ipadeola, M. Meremiku

**Affiliations:** ^1^Department of Population, Family and Reproductive Health, School of Public Health, University of Ghana, P.O. Box LG 25, Legon, Accra, Ghana; ^2^Planning, Research and Statistics Directorate, National Agency for Food and Drug Administration and Control, PMB 5023, Wuse, Abuja, Nigeria; ^3^PLAN-Health Project, Management Sciences for Health, P.O. Box 240, Kubwa, Abuja, Nigeria; ^4^Technical Services Directorate, Society for Family Health, PMB 5116, Wuse, Abuja, Nigeria; ^5^Society for Family Health, PMB 5116, Wuse, Abuja, Nigeria; ^6^Malaria Action Program for States, PMB 44, Abuja, Nigeria; ^7^University of Calabar, PMB 1115, Calabar, Nigeria

## Abstract

*Background*. Malaria during pregnancy is a major public health problem in Nigeria especially in malaria-endemic areas. It increases the risk of low birth weight and child/maternal morbidity/mortality. This paper addresses the impact of radio campaigns on the use of insecticide-treated bed nets among pregnant women in Nigeria. * Methods*. A total of 2,348 pregnant women were interviewed during the survey across 21 of Nigeria's 36 states. Respondents were selected through a multistage sampling technique. Analysis was based on multivariate logistic regression. * Results*. Respondents who knew that sleeping under ITN prevents malaria were 3.2 times more likely to sleep under net (OR: 3.15; 95% CI: 2.28 to 4.33; *P* < 0.0001). Those who listened to radio are also about 1.6 times more likely to use ITN (OR: 1.56; 95% CI: 1.07 to 2.28; *P* = 0.020), while respondents who had heard of a specific sponsored radio campaign on ITN are 1.53 times more likely to use a bed net (*P* = 0.019). * Conclusion*. Pregnant women who listened to mass media campaigns were more likely to adopt strategies to protect themselves from malaria. Therefore, behavior change communication messages that are aimed at promoting net use and antenatal attendance are necessary in combating malaria.

## 1. Background

Malaria during pregnancy is a major public health problem; it increases the risk of low birth weight (LBW) (<2500 g) and child morbidity and mortality during the first year of life by inducing intrauterine growth retardation, prematurity, infant anaemia, and maternal anaemia [1–3]. It is estimated that over 30 million women living in malaria-endemic parts of Africa become pregnant every year [4]. Malaria in pregnancy is responsible for 11% of maternal deaths in Nigeria [5]. In areas where malaria is endemic, 20–40% of all babies born may have low birth weight [6]. Malaria in pregnancy is a priority area in the Global Roll Back Malaria (RBM) strategy. In 2000, the World Health Organization recommended a package of interventions to prevent malaria during pregnancy. This package includes the promotion of insecticide-treated bed nets (ITNs), intermittent preventive treatment in pregnancy (IPT), and effective case management of malarial illness [7]. The strategy also promotes the integration of malaria prevention into the safe motherhood package. Therefore, one of the strategies of the Nigerian National Strategic Plan for Malaria Control is to create awareness on prevention of malaria in pregnancy through the use of ITNs, among others.

There has been a massive effort to ensure availability of ITNs in every household in Nigeria as part of the scaling up for impact (SUFI) which endeavours to ensure universal access to long lasting insecticide-treated nets (LLINs). During distribution campaigns, LLINs are provided free to household members such that every household has at least two nets. In addition, nets are provided at highly subsidized rates through social marketing in health facilities and drug stores for ease of access to pregnant women and women with children less than five years. The cost-effectiveness of ITNs relative to other forms of malaria prevention and treatment also makes it a better option for malaria prevention especially among residents of rural communities, people with poor access to health facilities, and people with low socioeconomic status [8–10]. The risk of malaria in pregnancy has been reported in several countries in sub-Saharan Africa including Uganda [11], Malawi [12], Tanzania [13], and Ethiopia [14].

In a study among pregnant women in Lagos state, only 11.2% and 37.5% actually used ITNs in two study hospitals [15]. In some parts of Nigeria, use of bed nets among pregnant women is still low. In a study in northern Nigeria, 73% of respondents have never used ITN before because of lack of awareness [16]. In another study in Ibadan, southwest Nigeria, only 20.9% of pregnant women use ITNs [17].

To promote use and uptake of these malaria prevention strategies, mass media campaigns have been initiated to sensitise the general public, particularly pregnant women, on the effectiveness and long term benefits of correct and consistent use of the ITNs during pregnancy. The mass media campaign messages were aired on national radio and television stations in English, Pidgin English, and the three main local languages in Nigeria. Also billboards with clear messages about the link between mosquitoes and malaria prevention were placed at strategic locations in major cities in Nigeria to further improve access to correct information. The messages on the billboards were reproduced into posters and handbills that were widely distributed across the country. This evaluation is focused only on the mass media campaign via the radio. Very few nationwide health interventions are evaluated. The aim of this paper is to assess the impact of exposure to these campaigns on uptake of the malaria prevention strategies in pregnancy.

## 2. Data and Methods 

### 2.1. Study Area and Population

Twenty-one out of the 36 states in Nigeria including the Federal Capital Territory were visited for this study. All the states were implementing programmes that draw from the National Strategic Plan for Malaria Control which is based on the principles of Global Malaria Control strategy and Roll Back Malaria initiatives. This study was a cross-sectional and population-based survey. A systematic multistage sampling technique was used to select pregnant women resident in the eighteen Global Fund states and three other adjoining states. The first stage was the selection of localities/sites to form 20 clusters. All the localities in a state were arranged in their geographic order with their sizes attached. The projected population of the localities was considered as the measure of size. A cumulative measure of size was obtained and using a systematic sampling procedure, 20 clusters were allocated to the localities. Pregnant women within the selected households were identified and screened for eligibility. Data were collected by trained interviewers using validated questionnaires to obtain information on maternal and child health with focus on malaria control. Data from a total of 2,348 pregnant women who were interviewed were collated and analysed.

The dependant variable is based on the responses from respondents to the question on whether the respondent slept under an ITN the night prior to the study. All analyses were performed using SPSS 18.0 and conclusions are based on the 0.05 level of significance.

Structured questionnaires based on thematic areas were administered by trained interviewers. The questionnaire was validated and pilot tested before use. Interviewers were trained with a specific focus on all aspects of the interview process and questionnaires. The questionnaires were pretested to check for comprehensibility of questions as well as procedures for conducting interviews. The study questionnaires elicited information on key programme indicators and the various issues related to knowledge, behaviour, and practices related to prevention and treatment of malaria. The questionnaire also elicited information to determine if respondents listened regularly to the radio and if they listened to specific messages presented in radio broadcasts that focused on prevention of malaria. Approval was also received from the State Ministries of Health and leaders of each of the communities selected for the survey. Informed verbal consent was obtained from the heads of households and the respondents; those who declined consent were not interviewed. All selected caregivers and pregnant women were interviewed in their homes and confidentiality was assured. During data collection, quality control processes were implemented by supervisors to identify and correct problems while in the field.

Data were entered using the Census and Survey Processing System (CSPro) and were exported to, managed, and analysed using Statistical Package for the Social Sciences (SPSS 18.0). Frequency tables and cross tabulations were used to generate data tables and figures to present results in keeping with survey objectives and analysis plan. We employed a logistic regression technique [18–20] to explore influence of factors such as knowledge about ITN, causes of malaria, and correct ways of preventing malaria on the use of bed nets.

Specifically, we explored exposure to mass media campaigns in Nigeria under the auspices of the National Malaria Control Programme with emphasis on messages promoting the use of ITNs. We aimed to determine whether the use of ITNs could be attributed to exposure to this mass media campaign. All categorical variables were dummy-coded. We explored the influence of these determinants on the use of ITNs and later explored the influence of exposure to mass media on the use ITNs. A binary dependent variable was created for use or nonuse of ITNs and determinants, as well as exposure to media. Statistical significance was based on a *P* value of 0.05. At an exploratory data analysis stage, several models were explored. Test of goodness-of-fit was based on Hosmer and Lemeshow test [21], with models with *P* > 0.05 assumed to be good and to fit well to the data. In this study, exposure was measured as having listened to radio and having heard of the special media campaigns on the use of ITNs. We proposed the null hypothesis that there will be no relationship between personal media exposure and use of ITNs.

## 3. Variables

Table 1 presents the general characteristics of the pregnant women involved in the survey. The dependent and independent variables are described in the next section.

### 3.1. Dependent Variable

The dependent variable is the use or nonuse of ITN the night immediately preceding the survey.

### 3.2. Independent

The key independent variables in this paper are geopolitical zones, residence, age, education, educational attainment, gestational stage of registration for antenatal care, knowledge about use of ITN, listenership to radio, exposure to campaigns on ITN, locality of residence (rural or urban), knowledge about modes of prevention of malaria, age of the respondents, and knowledge that mosquito bites are the main cause of malaria. All the independent variables were dummy-coded. For instance, five dummy variables were created for Nigeria's six geopolitical zones (northeast, northwest, southeast, southwest, and south-south) with north-central being the reference category. For locality of residence, rural area was considered as reference category, and respondents below age of 20 were considered as reference category for respondents' age. Education was an ordinal variable, with five possible responses regarding the highest level of education attained: no formal education, Qur'anic, primary, secondary, and postsecondary education.

## 4. Results

A total of 2,348 pregnant women were interviewed during the survey across the 21 states. Table 1 presents findings of the descriptive summary and sociodemographic characteristics of respondents. Overall, about a fifth of the respondents had never attended school. There is a substantial geographic variation with northeast having the highest proportion of respondents with no formal education. Respondents in age group 26 years and above constituted the largest proportion. About 37 percent of the respondents interviewed were housewives, 32 percent were self-employed, and only about 6 percent have a formal job (blue collar job). Unlike in the other geopolitical zones, a substantially larger proportion of the respondents were in urban areas than in rural areas.

Table 2 presents findings of the bivariate analyses of use of ITNs by selected characteristics. Knowledge of modes of preventing malaria, ownership of bed nets, knowledge that using an ITN prevents malaria, knowledge of dangers of malaria in pregnancy, and ANC attendance were all significantly associated with ITN use.

Table 3 presents findings from logistic regression model for use of ITNs. At an exploratory data analysis stage, several models were explored. However, we present the results for the model with best fit. Test of goodness-of-fit was based on Hosmer and Lemeshow test (HL). The test showed that the model presented here fits well to the data (*P* = 0.944). Exposure to mass media was observed to be positively associated with sleeping under ITN as those who listened to radio (OR = 1.56; 95% confidence interval 1.07 to 2.28; *P* = 0.02) and had heard of the mass media campaign (OR = 1.53; 95% confidence interval 1.07 to 2.17; *P* = 0.02) were about two times more likely to sleep under a bed net the night preceding the survey compared with their counterparts who did not. The knowledge that sleeping under ITN prevents malaria was significantly associated with sleeping under a bed net. Respondents with correct knowledge on use of ITNs were approximately three times more likely to sleep under ITNs compared with their counterparts who did not know that ITNs protect against malaria (OR = 3.15; *P* < 0.0001). Findings from this model also revealed a positive association between receiving IPT and use of ITN with those who received IPT about one and a half times more likely to sleep under ITNs (OR = 1.48; *P* = 0.02). However, the significant effect of registering for ANC which was evident in the bivariate model disappeared after adjusting for other covariates. This implies that the net effect of ITN use by registration for ANC cancelled out after adjusting for other covariates.

## 5. Discussion

The role of mass media cannot be overemphasised in addressing reproductive health issues particularly in view of its potential for wide audience reach and cost-effectiveness in reaching large audiences. The use of ITNs during pregnancy has been shown in a systematic review to have a beneficial impact on pregnancy outcome in malaria-endemic Africa [22]. Although bed nets are available at most health facilities, some pregnant women still encounter difficulties accessing the nets [16].

Thus the promotion of net use is critical in the quest for the eradication of malaria in Africa. Combining the use of bed nets and IPT is perhaps the surest way of preventing malaria in pregnant mothers and this will in turn promote the health of mothers and their babies. Promoting use of ITNs, through mass media, has proved to be effective and has improved ITN use among pregnant women in Nigeria. ITNs when used by a large number of the target population within a community are effective not only in preventing malaria but also in generating a residual effect by significantly reducing vector populations thereby reducing rate of transmission [23], though this is presently still being discussed in the literature [24]. In addition the production of education and communication materials on malaria in pregnancy to be expanded and distributed nationally to all antenatal clinics in both private and government facilities, particularly in rural areas.

Although there have been increased efforts on the distribution of ITNs and education on their use in Nigeria, ITN ownership is still low as at the time of this survey. Reasons for poor use include misconceptions about ITNs and poor knowledge about the efficacy of ITNs in preventing malaria. There are still several misconceptions about malaria that negatively affects the number of pregnant women who use bed nets [25]. Efforts should be directed at uptake of antenatal services as soon as a woman confirms her pregnancy status. This will avail her opportunity of receiving ITNs (which are often distributed at no cost in most antenatal facilities) for the benefit of both mother and child. The low level of utilisation of ITNs has led to the call to expand the involvement of mass media [26] and the results of our study provide some evidence to support this call. This paper will guide policy makers and stakeholders in the planning and implementation of intervention in maternal and child health to adopt the mass media and an effective approach to reaching target groups and the general population.

## 6. Conclusion 

The use of mass media in promoting the use of bed nets is effective. Respondents who knew that sleeping under ITN prevents malaria were 3.2 times more likely to sleep under net (OR: 3.15; 95% CI: 2.28 to 4.33; *P* < 0.0001). Those who listened to radio are about 1.6 times more likely to use ITN (OR: 1.56; 95% CI: 1.07 to 2.28; *P* = 0.020), while respondents who had heard of a specific (monitored) sponsored radio campaign on ITN are 1.53 times more likely to use a bed net (*P* = 0.019). As much as one would have expected the respondents who have attained secondary education or higher to use net, findings showed a reverse relationship, although this was not statistically significant. The level of utilization of ITNs in Nigeria is still low. We advocate for expanded involvement of the mass media in community enlightenment programmes. Furthermore, efforts at the distribution of ITNs in all antenatal clinic outlets should be intensified.

## 7. Limitation of Study

In spite of the findings from this study, it is worth noting that a similar challenge of inability to establish causal relationship as in any cross-sectional study was encountered in this study. Further, the survey did not include questions to measure socioeconomic status of the respondents which are possible confounders to subject of interest. Therefore, we recommend that results be interpreted with an element of caution.

## Figures and Tables

**Table 1:** Percentage distribution of the demographic characteristics of the respondents included in the survey.

Characteristics	Urban (*n* = 1064)		Rural (*n* = 1284)		Total (*n* = 2348)	
	*n*	%	*n*	%	*n*	%
Age of respondent						
Below 26 years	331	31.1	505	39.3	836	35.6
26 years and above	733	68.9	779	60.7	1512	64.4
Education						
None	137	12.9	316	24.6	453	19.3
Qur'anic only	114	12.3	208	21.5	322	13.7
Primary	210	22.7	326	33.7	536	22.8
Secondary	466	50.3	369	31.8	835	35.6
Higher	137	14.8	65	6.7	202	8.6
Geopolitical zones						
Northwest	197	18.5	260	20.2	457	19.4
Northeast	184	17.3	268	20.9	452	19.3
North-central	189	17.8	263	20.5	452	19.3
Southwest	247	23.1	101	7.9	348	14.8
Southeast	110	10.3	188	14.6	298	12.7
South-south	137	12.9	204	15.9	341	14.5
Occupation						
Formal job	87	8.2	51	3.9	138	5.9
Self-employed	366	34.4	387	30.2	753	32.0
Housewives	397	37.3	481	37.5	878	37.4
Others	248	22.3	331	29.3	579	24.7
ANC attendance						
Registered	618	58.1	630	49.1	1248	53.2
Did not register	446	41.9	654	50.9	1100	46.8
Ownership of nets						
Yes	296	34.8	381	35.6	677	35.3
No	557	65.3	1114	64.3	1671	64.7
Received IPT						
Yes	247	23.2	282	22	529	22.5
No	817	76.8	1002	78	1819	77.5

**Table 2:** Bivariate analysis showing percentage distribution of use of ITN according to selected sociodemographic characteristics.

Characteristics	Use of ITN				
	Yes (*n* = 259)		No (*n* = 2089)		*P* value
	Value	%	Value	%	
Locality					
Rural	126	11.8	938	88.2	0.141
Urban	133	10.4	1151	89.6	
Age of caregivers					
Below 26 years	91	10.9	745	89.1	0.463
26 years and above	168	11.1	1344	88.9	
Education					
None	46	10.2	407	89.8	0.268
Qur'anic only	37	11.5	285	88.5	
Primary	69	12.9	467	87.1	
Secondary	80	9.6	755	90.4	
Higher	27	13.4	175	86.6	
Geopolitical zones					
Northwest	71	15.5	366	84.5	0.016
Northeast	47	10.4	405	89.6	
North-central	49	10.8	403	89.2	
Southwest	27	7.8	321	92.2	
Southeast	31	10.4	267	89.6	
South-south	34	10.0	307	90.0	
Registered for ANC					
No	98	8.9	1002	91.1	0.001
Yes	161	12.9	1087	87.1	
Knowledge of dangers of malaria in pregnancy					
No	208	11.3	1638	88.7	0.269
Yes	51	10.2	451	89.8	
Knowledge that ITN prevents malaria					
No	134	7.6	1619	92.4	<0.001
Yes	125	21.2	464	78.8	
Ownership of a bed net					
No	5	0.3	1666	99.7	<0.001
Yes	254	37.5	423	62.5	
Knowledge of causes of malaria					
No	134	9.9	1220	90.1	0.024
Yes	125	12.6	869	87.4	
Risk of malaria in pregnancy					
Perceived it as not harmful	16	6.5	229	93.5	0.017
Perceived it as harmful	242	11.6	1847	88.4	
Misconception about causes of malaria					
No misconception	139	12.0	1017	88.0	0.074
Had misconception	120	10.1	1072	89.9	
Misconception about prevention of malaria					
No misconception	124	10.2	1093	89.8	0.100
Had misconception	135	11.9	996	88.1	
Knowledge of modes of prevention of malaria					
Not correct knowledge	147	8.5	1576	91.5	<0.001
Correct knowledge	112	17.9	513	82.1	
Total	**259**		**2089**		

**Table 3:** Results from logistic regression model 1 with respondents who slept under bed net as outcome variable.

Variable	Odds ratio	*P* value	Confidence level	
			Lower	Upper
Northwest	1.53	0.07	0.96	2.45
Northeast	0.87	0.60	0.51	1.49
Southwest	0.98	0.95	0.57	1.69
Southeast	1.19	0.51	0.71	2.01
South-south	1.41	0.19	0.85	2.33
Secondary and higher education	0.74	0.06	0.53	1.02
Registered for ANC	1.19	0.31	0.85	1.65
Knew that using ITN prevents malaria	3.15	0.00	2.28	4.33
Listened to radio	1.56	0.02	1.07	2.28
Heard of campaign on ITN	1.53	0.02	1.07	2.17
Received IPT	1.48	0.02	1.06	2.08
